# Drug Use Patterns in Wastewater and Socioeconomic and Demographic Indicators

**DOI:** 10.1001/jamanetworkopen.2024.32682

**Published:** 2024-09-23

**Authors:** Xiaowei Zhuang, Michael A. Moshi, Oscar Quinones, Rebecca A. Trenholm, Ching-Lan Chang, Dietmar Cordes, Brett J. Vanderford, Van Vo, Daniel Gerrity, Edwin C. Oh

**Affiliations:** 1 Laboratory of Neurogenetics and Precision Medicine, University of Nevada Las Vegas; 2 Neuroscience Interdisciplinary PhD Program, University of Nevada, Las Vegas; 3 Cleveland Clinic Lou Ruvo Center for Brain Health, Las Vegas, Nevada; 4 Applied Research and Development Center, Southern Nevada Water Authority, Las Vegas; 5 Department of Brain Health, University of Nevada, Las Vegas; 6 Department of Internal Medicine, Kirk Kerkorian School of Medicine, University of Nevada, Las Vegas

## Abstract

**Importance:**

Measuring drug use behaviors in individuals and across large communities presents substantial challenges, often complicated by socioeconomic and demographic variables.

**Objectives:**

To detect spatial and temporal changes in community drug use by analyzing concentrations of analytes in influent wastewater and exploring their associations with area-based socioeconomic and sociodemographic metrics like the area deprivation index (ADI) and rural-urban commuting area (RUCA) codes.

**Design, Setting, and Participants:**

This longitudinal, cross-sectional wastewater study was performed from May 2022 to April 2023 and included biweekly influent wastewater samples of 39 analytes from 8 sampling locations across 6 wastewater treatment plants in southern Nevada. Statistical analyses were conducted in December 2023.

**Main Outcomes and Measures:**

It was hypothesized that wastewater monitoring of pharmaceuticals and personal care products (PPCPs) and high-risk substances (HRSs) could reveal true spatial and temporal drug use patterns in near–real time. Data collection of samples for PPCPs and HRSs was performed using mass spectrometry. Both ADI and RUCA scores were utilized to characterize neighborhood contexts in the analysis. The false discovery rate (FDR) method was utilized to correct for multiple comparisons (*P*_FDR_).

**Results:**

Over the 12-month wastewater monitoring period, 208 samples for PPCPs and HRSs were collected, and analysis revealed an increase in the consumption of HRSs and the seasonal variation in PPCP use in southern Nevada. There was a significant increase in levels of stimulant-associated analytes, such as cocaine (β = 9.17 × 10^−4^; SE = 1.29 × 10^−4^; *P*_FDR_ = 1.40 × 10^−10^), and opioids or their metabolites, notably norfentanyl (β = 1.48 × 10^−4^; SE = 1.88 × 10^−4^; *P*_FDR_ = 1.66 × 10^−12^). In contrast, DEET, an active ingredient in mosquito and tick repellents, demonstrated a seasonal use pattern (β = −4.85 × 10^−4^; SE = 2.09 × 10^−4^; *P*_FDR_ = 4.87 × 10^−2^). Wastewater from more disadvantaged or rural neighborhoods, as assessed through ADI and RUCA scores, was more likely to show a significant positive correlation with HRSs, such as cocaine (β = 0.075; SE = 0.038; *P* = .05) and norfentanyl (β = 0.004; SE = 0.001; *P* = 1.64 × 10^−5^).

**Conclusions and Relevance:**

These findings suggest that wastewater monitoring of PPCPs and HRSs offers a complementary method to existing public health tools, providing timely data for tracking substance use behaviors and use of PPCPs at a population level.

## Key Points

**Question:**

Can drug consumption behaviors in southern Nevada communities be characterized through wastewater monitoring data, and are such data associated with sociodemographic and socioeconomic metrics?

**Findings:**

In this cross-sectional study conducted from May 2022 to April 2023, 208 wastewater samples for 39 analytes were collected from treatment plants serving approximately 2.4 million residents and 50 million annual visitors to southern Nevada (ie, Las Vegas). The analysis showed unique patterns in drug use that varied by community and neighborhood and over time, including changes in the use of personal care products depending on the season and a general rise in the use of high-risk substances.

**Meaning:**

These findings suggest that monitoring drug compounds and metabolites in wastewater can reveal significant use patterns that are influenced by the characteristics of distinct population groups.

## Teaser

This cross-sectional study examines associations of concentrations of drug analytes in wastewater with socioeconomic and sociodemographic metrics in southern Nevada.

## Introduction

Monitoring drug consumption behaviors in the US presents a complex challenge, both at individual and community levels.^1,2^ Individuals often hesitate to self-report due to a variety of concerns encompassing social stigma, ethical dilemmas, privacy issues, and legal ramifications.^3^ This reluctance can lead to biases that diminish the accuracy and reliability of collected data. At the community level, drug consumption behaviors are subject to rapid changes, often influenced by the emergence of new substances and the prevalence of polydrug use.^4,5^ Furthermore, neighborhood characteristics—such as the degree of urbanization, demographic profiles, and social determinants of health—can substantially alter drug consumption patterns.^6^ Historically, neighborhood disparities have been associated with various health-related behaviors, outcomes, and mortality,^7,8,9^ yet the specific impact of urbanization and social determinants on drug consumption patterns remains an underexplored area. A deeper understanding of the interplay between drug consumption behaviors and socioeconomic factors could aid in identifying risk factors for drug overdoses and support efforts to promote health equity.

In response to COVID-19, wastewater monitoring programs have gained renewed importance as a method for tracking public health threats, providing real-time insights through the analysis of community sewage.^10,11,12^ This method can detect a wide range of substances, from pharmaceuticals^2,13,14^ to pathogens,^15,16,17,18,19,20,21^ thereby reflecting the health behaviors and exposures of a population. Such analysis reveals important trends in drug use, dietary habits, and the presence of environmental contaminants—key indicators of social determinants like economic status, health care access, and environmental risks. Moreover, it can uncover health disparities across neighborhoods by examining substance concentrations that correlate with socioeconomic and lifestyle factors. Studies outside the US, for instance, have shown an association of socioeconomic or sociodemographic factors with the consumption of specific chemicals or dietary components.^6^ In the US, tools like the area deprivation index (ADI)^22,23^ and rural-urban commuting area (RUCA) codes^24^ provide in-depth insights into these factors. When used in conjunction with wastewater analytics, these tools have the potential to enable a detailed understanding of health disparities across neighborhoods.

In this study, we analyzed wastewater data from southern Nevada over a span of 12 months to characterize drug consumption behaviors across a population of approximately 2.4 million people and the approximately 50 million tourists that visit Las Vegas annually. Using wastewater data on high-risk substances (HRSs) and pharmaceuticals and personal care products (PPCPs), we asked several questions: (1) Do drug consumption patterns cluster based on geographic locations? (2) Do consumption patterns change over time? (3) Are socioeconomic variables associated with the consumption of HRSs and PPCPs? Taken together, our data highlight how wastewater data can be used to complement conventional public health tools and be leveraged for the analysis of population health dynamics in southern Nevada.

## Methods

### Data Source

This longitudinal, cross-sectional study adhered to the Strengthening the Reporting of Observational Studies in Epidemiology (STROBE) reporting guideline.^25^ The University of Nevada Las Vegas institutional review board reviewed this project and determined it to be exempt from human participant research regulations and the requirement of informed consent according to federal regulations and university policy.

### Wastewater Collection and Analysis

**Figure 1.**
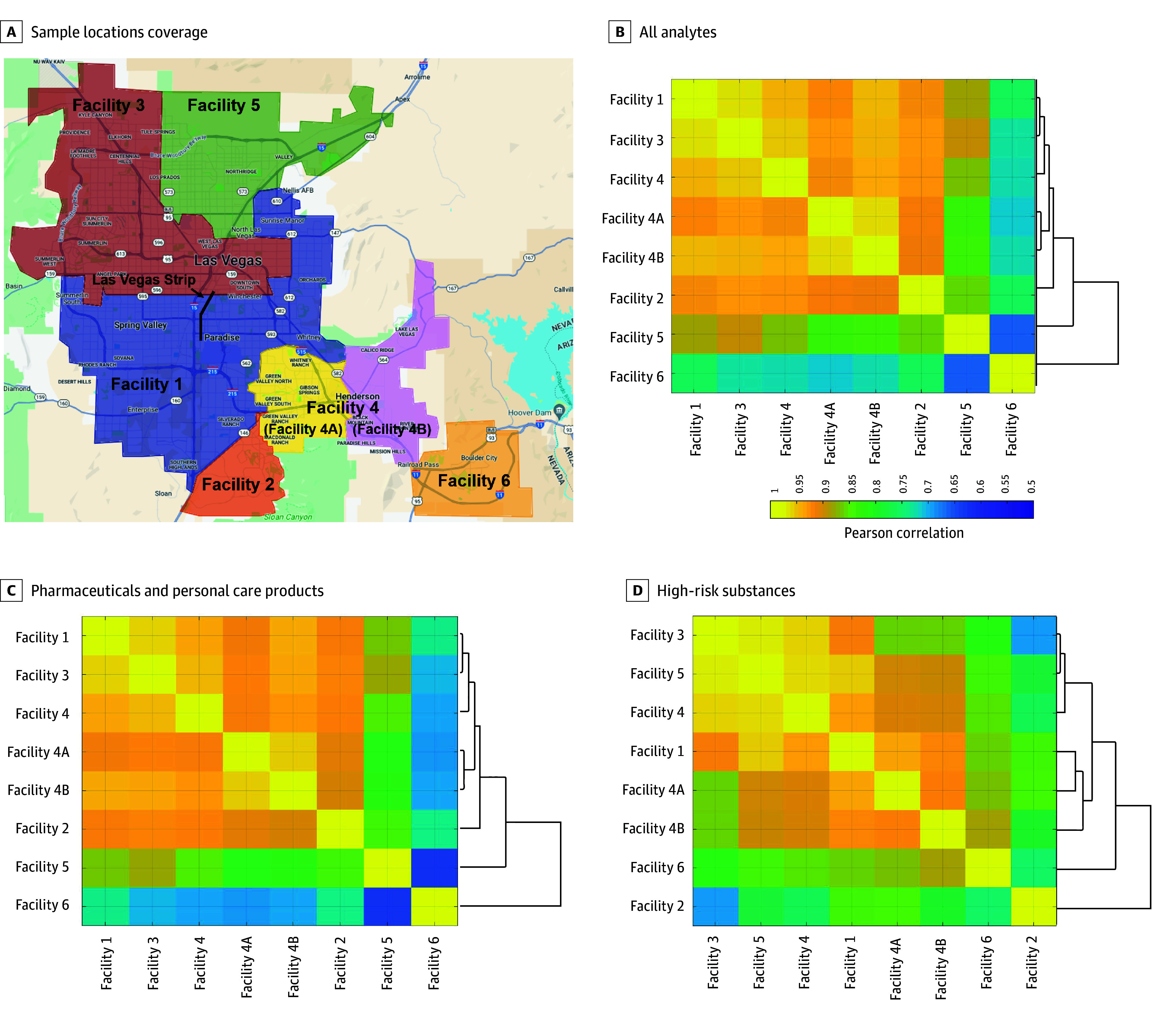
Spatial Characteristics of Pharmaceutical and Personal Care Products (PPCPs) and High-Risk Substance (HRS) Use Patterns Across Southern Nevada Sewersheds From May 2022 to April 2023 A, Map of 8 sampling locations (ie, sewersheds) in southern Nevada. The Las Vegas Strip is shown for reference. Similarities in sewershed-specific use patterns were based on the Pearson correlation matrix for all PPCPs and HRSs combined (B; 884 measurements for each location), PPCPs only (C), and HRSs only (D). Use patterns were based on wastewater concentrations normalized by flow and population (mg/person-day).

For this study, the methodology for wastewater collection and analysis by liquid chromatography tandem mass spectrometry with isotope dilution was extensively detailed in Gerrity et al.^14^ Briefly, from May 2022 to April 2023, we collected wastewater samples every 2 weeks from 8 sampling locations, each representing a distinct sewershed (ie, community), across 6 wastewater treatment plants. The corresponding sewersheds are delineated in Figure 1A and eTable 1 in Supplement 1. Facility 4 is a 24-hour composite sample for the combined sewershed spanning facility 4A and facility 4B. Facilities 4A and 4B were also independently monitored using grab samples collected from the respective sewer trunk lines prior to their entry into facility 4 because the trunk lines are not equipped with autosamplers capable of collecting composite samples.

These samples were collected directly into amber glass vials containing 50 mg/L of ascorbic acid (for oxidant quenching, albeit not needed for this study) and 1 g/L of sodium azide (for biological preservation)^14^ Samples were briefly stored at 4 °C prior to processing and analysis, typically within 1 to 2 days, and the target analytes included 17 PPCPs and 22 HRSs, including major metabolites. The HRSs were the primary focus of this study, but the PPCPs were also included to provide a point of comparison between commonly used licit substances vs illicit substances. In addition, Gerrity et al^14^ demonstrated that some PPCPs (eg, sucralose) have value in terms of normalizing HRS concentrations to account for fluctuations in the urine or fecal content of wastewater samples. Sample processing and analysis for PPCPs included automated solid phase extraction and injection of methanol extracts, while HRS analysis involved direct injection of 10-fold diluted aqueous samples. PPCP analysis was conducted using an atmospheric pressure ionization 4000-series mass spectrometer (SCIEX), employing both negative and positive electrospray ionization in multiple reaction monitoring mode. Drug analytes were tested on a 6500-quadrupole ion trap mass spectrometer (SCIEX), focusing on positive electrospray ionization in multiple reaction monitoring mode. Detailed methods were described previously in Gerrity et al^14^ and are also included in the eMethods and eTables 2 to 4 in Supplement 1.

Five analytes, including Δ-9-tetrahydrocannabinol (THC); heroin; 3,4-Methylenedioxyamphetamine (MDA); norcocaine; and triclocarban, were detected in fewer than 10% of the samples in this study, so our analysis focused on the remaining 34 analytes (16 PPCPs and 18 HRSs). This was in part due to aqueous instability (eg, THC and heroin) and/or insufficient sensitivity (eg, MDA and norcocaine) for certain analytes. However, the target compound list included other relevant analytes that could inform consumption patterns for the parent compounds of interest. For example, the major metabolites 11-nor-9-THC (THC-COOH) and 11-hydroxy-THC (THC-OH) were used to assess THC consumption; the major metabolite 6-acetylmorphine was used to assess heroin use; and cocaine, benzoylecgonine, ecgonine methyl ester, and ecgonine served as alternatives to norcocaine. 3,4-Methylenedioxymethamphetamine (MDMA) is sufficiently stable as a parent compound to assess consumption directly (ie, rather than using MDA). Finally, triclocarban was banned by the US Food and Drug Administration in September 2016.^26^

### Statistical Analysis

#### Unsupervised Clustering

As proof of concept that wastewater drug concentrations can be a reliable source of drug use patterns, we conducted unsupervised clustering analyses on use patterns across facilities or drugs. To evaluate PPCP and HRS use patterns across facilities, 26 distinct time points for all 34 analytes were assessed as features for each facility. We developed a similarity matrix for the facilities by calculating pairwise Pearson correlations, followed by hierarchical clustering to determine the similarities and differences among them.

For an unbiased evaluation of the interconnections between different use patterns, we carried out a separate hierarchical clustering analysis on the analytes, considering their concentrations at 26 time points across all 8 sampling locations. This approach helped establish similarities among analytes based on Pearson correlation measures. We applied this methodology for both PPCPs and HRSs, enabling a comprehensive analysis of their respective use patterns. Due to the potential sensitivity of the data, the 8 locations representing distinct communities were randomly anonymized (ie, Facilities A through H) for the following analyses to protect the privacy of the sampled populations.

#### Spatial and Temporal Analyses: Linear Mixed-Effects Model

To assess both temporal changes and spatial differences across facilities for each analyte, we employed a linear mixed-effects (LME) model. This model incorporated fixed effects for the location (8 sampling locations), time (26 time points), and their interaction (location × time). Random effects included the intercept and time variation by location. To account for multiple comparisons (N_drug_ ×  3), we applied a false discovery rate (FDR) correction method (*P*_FDR_) to the raw *P* values for both main and interaction effects. The statistical significance level was established at *P*_FDR_ ≤ .05.

To explore whether use patterns vary based on population background and neighborhood context, our focus was on analytes showing significant location effects or interaction effects in the LME model. Post hoc 2-sample *t* tests (2-sided) between facilities B vs G and facilities B vs H were conducted to analyze differences in use patterns in association with ADI and RUCA code variations, respectively. In addition, for these analytes, another LME model was utilized to examine the association of use patterns with ADI scores for all sampling locations, calculated as the mean ADI across all zip codes represented by a given location. Here, we used individual analyte concentrations at each time point, rather than temporal means, to enhance statistical power. The fixed effects in this second model were ADI and time, while the random effects remained consistent with the first model.

All statistical analyses were conducted in MATLAB version 2022b (Mathworks). Data analysis was conducted in December 2023.

## Results

### Drug Use Patterns Across Wastewater Facilities in Southern Nevada

For each facility, we calculated flow- and population-normalized concentrations (ie, units of mg/person-day) for 34 analytes across 26 time points, resulting in a total of 884 measurements. Our unsupervised clustering results demonstrated that pairwise Pearson correlations (*r*) of drug use among these facilities consistently exceeded 0.90, indicating significant similarities in use trends (Figure 1B). The highest similarity was found between Facilities 1 and 3 (*r* = 0.96), both serving larger populations, and between facilities 4A and 4B (*r* = 0.96), representing geographically adjacent communities. In contrast, when compared with other facilities, facility 5 (mean [SD] correlation of patterns with other facilities, *r* = 0.83 [0.08]) and facility 6 (mean [SD] correlation of patterns with other facilities, *r* = 0.72 [0.03]) displayed distinct patterns (Figure 1B). Although the patterns for PPCPs mirrored the overall trends for PPCPs combined with HRSs (Figure 1C), the HRS-focused analysis showed significant differences, especially in facilities 2 and 6 compared with other wastewater treatment plants (Figure 1D).

**Figure 2.**
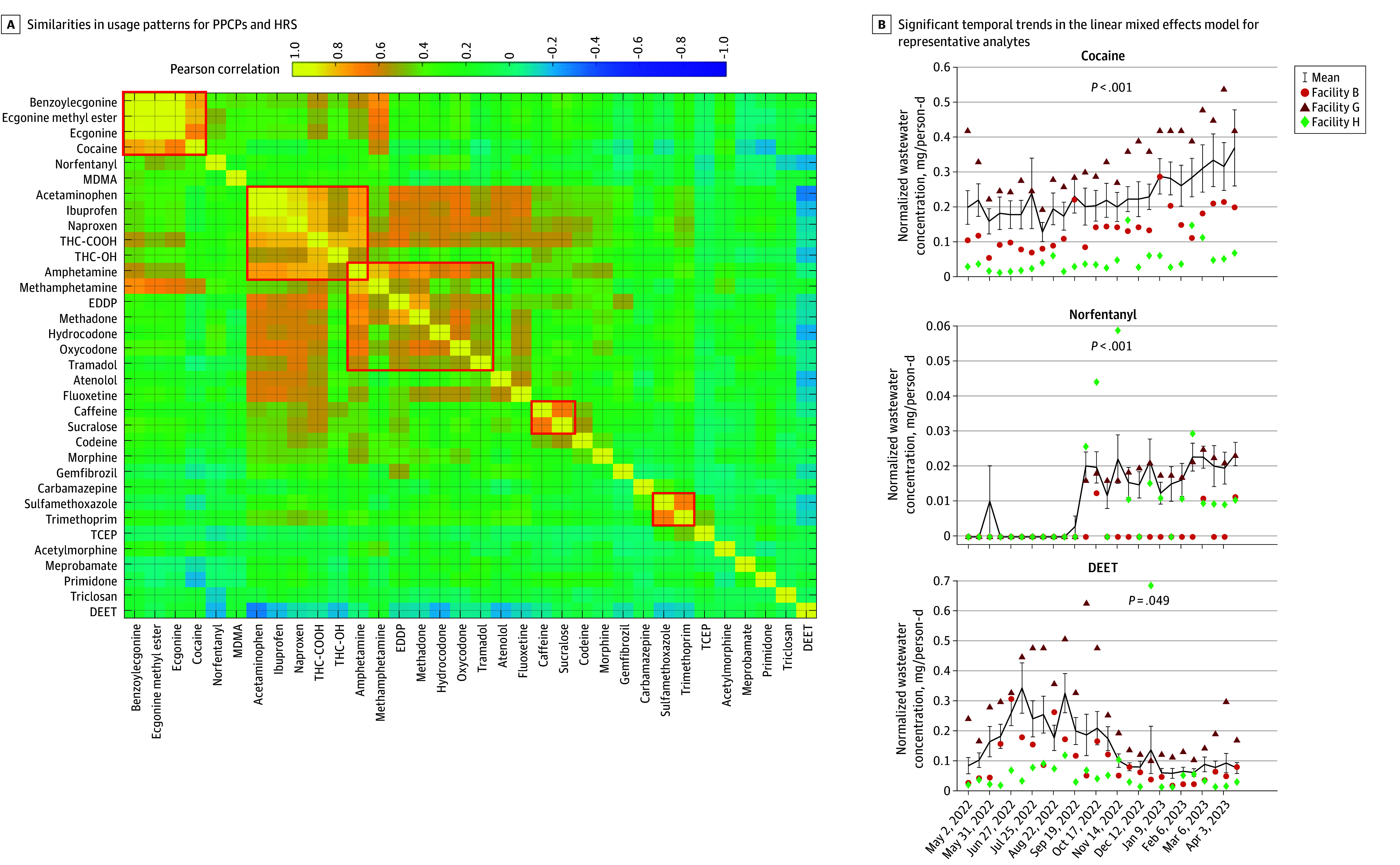
Correlations and Temporal Characteristics of Pharmaceuticals and Personal Care Products (PPCPs) and High-Risk Substances (HRSs) in Southern Nevada Sewersheds From May 2022 to April 2023 A, Similarities in use patterns for PPCPs and HRSs across all sewersheds, revealed by the interanalyte Pearson correlation matrix (208 measurements for each analyte). Five red boxes, from upper left to bottom right, indicate similar use and occurrence patterns of (1) cocaine-related metabolites, (2) pain relievers, (3) opioids, (4) food additives, and (5) prescribed antibiotics. B, Significant temporal trends in the linear mixed-effects model for representative analytes, including cocaine, norfentanyl (major metabolite of fentanyl), and seasonal use of N-diethyl-meta-toluamide (DEET). Note that sewersheds were randomly anonymized as facilities A through H, and use patterns were based on wastewater concentrations normalized by flow and population (mg/person-day). EDDP indicates 3-diphenylpyrrolidine; MDMA, 3,4-Methylenedioxymethamphetamine; TCEP, tris (2-chloroethyl) phosphate; THC-COOH, 11-nor-9-carboxy-Δ-9-tetrahydrocannabinol; THC-OH, 11-hydroxy-Δ-9-tetrahydrocannabinol.

As a validation of our approach, we characterized the similarities for each analyte across 26 time points and 8 sampling locations. We found a robust correlation in the levels of cocaine and its 3 of its 4 metabolites, specifically ecgonine, ecgonine methyl ester, and benzoylecgonine (total of 208 measurements for each analyte; mean [SD] *r* = 0.87 [0.09]) (Figure 2A). Pain relievers, including acetaminophen and the 2 nonsteroidal anti-inflammatory drugs (NSAIDs; ibuprofen and naproxen), recreational marijuana metabolites (THC-COOH and THC-OH), and central nervous system (CNS) stimulants (amphetamine and methamphetamine) also showed closely correlated use patterns (mean [SD] *r* = 0.74 [0.12]). Opioids such as methadone (and its major metabolite, 2-ethylidene-1,5-dimethyl-3,3-diphenylpyrrolidine [EDDP]), oxycodone, hydrocodone, and tramadol exhibited similar consumption trends (mean [SD] *r* = 0.58 [0.12]) and were correlated with the use of the over-the-counter pain relievers. Additionally, our observations revealed interconnected use patterns among specific PPCPs. This is highlighted by the significant correlation between caffeine and sucralose (*r* = 0.67), as well as the frequent coprescription of certain antibiotics, such as sulfamethoxazole and trimethoprim (*r* = 0.69) (Figure 2A).

### Temporal and Spatial Trends in Drug Use Patterns

With repeated measurements of PPCP- and HRS-related analytes across 8 facilities, we sought to identify both temporal and spatial trends in use and consumption patterns using LME models. Please note that we anonymized the 8 sampling locations for the following analyses; therefore, facility designations in the subsequent discussion are different from those presented in Figure 1.

#### Temporal Trends

We discovered a significant time-effect in the LME model for 9 of the 34 analytes (Figure 2B and eFigure 1 and eTable 5 in Supplement 1). Among the HRSs, cocaine occurrence exhibited a steady rise over the duration of the study (β = 9.17 × 10^−4^; SE = 1.29 × 10^−^4; *P*FDR = 1.40 × 10^−10^), as did its major metabolites ecgonine, ecgonine methyl ester, and benzoylecgonine, indicating a marked increase from 2022 to 2023 (Figure 2B and eFigure 1 in Supplement 1). Prior to September 2022, norfentanyl was detected above the method reporting limit only on Memorial Day weekend in May 2022 at Facility E, but a significant increase in detection frequency (ie, above the method reporting limit) and concentration was observed at all facilities starting in September and October 2022 (β = 1.48 × 10^−4^; SE = 1.88 × 10^−4^; *P*_FDR_ = 1.66 × 10^−12^) (Figure 2B). Furthermore, a direct comparison of analyte concentrations for facility 1 suggested an increase in consumption between 2010^13^ and 2023 for 14 HRSs (eTable 6 in Supplement 1).

Unlike for the HRSs, the use patterns of PPCPs showed significant temporal fluctuations between 2022 and 2023. N,N-diethyl-meta-toluamide (DEET), an insect repellent ingredient, peaked in the summer of 2022 and declined toward spring 2023 (β = −4.85 × 10^−4^; SE = 2.09 × 10^−4^; *P*_FDR_ = 4.87 × 10^−2^) (Figure 2B), reflecting a seasonal use pattern. Acetaminophen use surged in November 2022, remaining high through the holiday season until January 2023 (β = 0.07; SE = 0.03; *P*_FDR_ = 3.36 × 10^−2^) (eFigure 1 in Supplement 1). The level of PPCP use in 2023 paralleled that in 2010^13^ (eTable 6 in Supplement 1), including atenolol, primidone, carbamazepine, trimethoprim, sulfamethoxazole, and DEET. Interestingly, a decline was observed in the use of meprobamate, a popular sedative in the 1950s, and occurrence of tris (2-chloroethyl) phosphate (TCEP), a flame retardant, compared with 2010 (eTable 6 in Supplement 1). Meprobamate is also a metabolite of carisoprodol, a popular muscle relaxant.^27^ Overall, our analysis highlights an uptick in HRSs consumption and seasonal variation in PPCP use and occurrence in southern Nevada.

#### Spatial Trends and Correlations Between PPCP and HRS Use Patterns and Neighborhood Context

**Figure 3.**
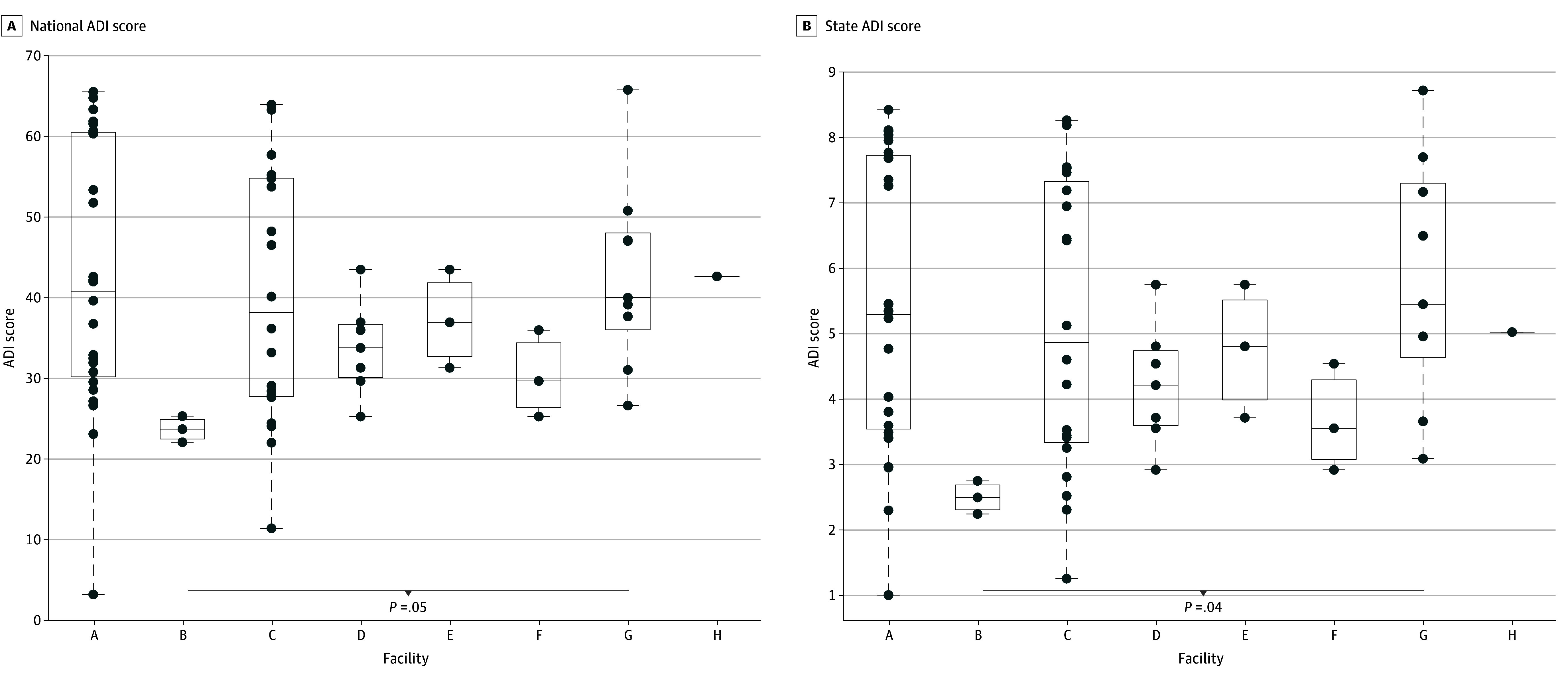
Boxplots of Socioeconomic Characteristics for Each Sewershed, Using the Area Deprivation Index (ADI) Scores The ADI allows for rankings of neighborhoods by socioeconomic disadvantage at both the national (national percentile, A) and state (state decile, B) levels. A higher ADI score indicates greater socioeconomic disadvantage. Sewersheds were randomly anonymized as facilities A through H. Facilities B and G were significantly different in both national percentile (*P* = .05) and state decile (*P* = .04).

Except for the antibiotics trimethoprim and sulfamethoxazole, the flame retardant TCEP, and the antimicrobial compound triclosan, the LME model showed a significant location or location × time effect in the LME model for nearly all analytes (eTable 5 in Supplement 1), highlighting distinct spatial trends across the facilities. We next sought to correlate these spatial use differences with neighborhood contexts. Our retrospective analysis of the urban-rural status and neighborhood contexts of 8 sampling locations revealed 2 key findings. First, all facilities except facility H shared a RUCA code of 1, indicating that they cover a metropolitan area core with primary flow within an urbanized area. Facility H was unique, with a higher RUCA code of 2, indicating a lower level of urbanization compared with the other facilities. Second, facility G had a significantly higher ADI than facility B, suggesting a more socioeconomically disadvantaged population (Figure 3). The significant post hoc pairwise differences between facilities B and H, and between facilities B and G further associated distinct analyte occurrence patterns with neighborhood contexts (Table). For HRSs, significant consumption pattern differences were observed between facilities, with facilities B and G typically showing the lowest and highest rates, respectively. In contrast, fewer PPCPs showed significant differences between facilities (Table).

**Table.** Post Hoc Comparisons Indicating Significant Differences in Normalized Wastewater Concentrations Based on ADI or RUCA Code for Facilities B, G, and H

Drug	Drug category	Normalized wastewater consumption , mean (SD), mg/person-day			*P* value^a^	
					Facilities with significant ADI difference (facility B vs facility G)	Facilities with RUCA differences (facility B vs facility H)
		Facility B	Facility G	Facility H		
Acetaminophen	PPCP	44.02 (12.40)	24.47 (9.13)	52.49 (16.19)	4.00 × 10^−8^	3.92 × 10^−2^
Ibuprofen	PPCP	7.28 (2.76)	5.94 (1.01)	8.69 (2.58)	2.42 × 10^−2^	NA^b^
Naproxen	PPCP	4.5 (1.37)	4.44 (0.69)	6.44 (1.41)	NA^b^	6.75 × 10^−6^
Atenolol	PPCP	0.60 (0.19)	0.32 (0.05)	0.34 (0.22)	2.54 × 10^−9^	4.38 × 10^−5^
Carbamazepine	PPCP	0.02 (0.01)	0.03 (0.01)	0.04 (0.06)	4.20 × 10^−4^	NA^b^
Gemfibrozil	PPCP	0.39 (0.08)	0.28 (0.11)	0.47 (0.56)	1.33 × 1^−-4^	NA^b^
Meprobamate	PPCP	0.05 (0.01)	0.04 (0.01)	0.09 (0.08)	3.51 × 10^−6^	1.26 × 10^−2^
Primidone	PPCP	0.08 (0.05)	0.06 (0.03)	0.11 (0.15)	4.46 × 10^−2^	NA^b^
Sulfamethoxazole	PPCP	NA^b^	NA^b^	NA^b^	NA^b^	NA^b^
Trimethoprim	PPCP	NA^b^	NA^b^	NA^b^	NA^b^	NA^b^
Fluoxetine	PPCP	NA^b^	NA^b^	NA^b^	NA^b^	NA^b^
Caffeine	PPCP	29.36 (6.5)	21.68 (3.83)	40.68 (40.81)	3.83 × 10^−6^	NA^b^
DEET	PPCP	0.10 (0.08)	0.27 (0.15)	0.07 (0.13)	2.34 × 10^−6^	NA^b^
Sucralose	PPCP	NA^b^	NA^b^	NA^b^	NA^b^	NA^b^
TCEP	PPCP	NA^b^	NA^b^	NA^b^	NA^b^	NA^b^
Triclosan	PPCP	NA^b^	NA^b^	NA^b^	NA^b^	NA^b^
Acetylmorphine	HRS	0 (0.01)	0.01 (0.01)	0.01 (0.01)	6.87 × 10^−3^	NA^b^
Codeine	HRS	0.04 (0.01)	0.05 (0)	0.05 (0.04)	6.70 × 10^−5^	5.26 × 10^−2^
EDDP	HRS	0.02 (0)	0.04 (0)	0.06 (0.03)	3.37 × 10^−17^	9.49 × 10^−8^
Hydrocodone	HRS	0.04 (0.01)	0.05 (0.01)	0.06 (0.03)	9.84 × 10^−6^	8.27 × 10^−4^
MDMA	HRS	NA^b^	NA^b^	NA^b^	NA^b^	NA^b^
Methadone	HRS	0 (0)	0.01 (0.01)	0.02 (0.01)	1.12 × 10^−8^	7.56 × 10^−22^
Morphine	HRS	0.13 (0.02)	0.21 (0.01)	0.37 (0.30)	2.18 × 10^−20^	1.99 × 10^−4^
Norfentanyl	HRS	0 (0)	0.01 (0.01)	0.01 (0.02)	2.14 × 10^−5^	1.03 × 10^−2^
Oxycodone	HRS	0.04 (0.01)	0.04 (0.01)	0.05 (0.02)	6.23 × 10^−4^	3.06 × 10^−4^
Tramadol	HRS	0.13 (0.02)	0.14 (0.01)	0.20 (0.06)	1.56 × 10^−3^	7.37 × 10^−7^
Amphetamine	HRS	0.11 (0.01)	0.15 (0.02)	0.20 (0.04)	1.18 × 10^−13^	4.22 × 10^−15^
Benzoylecgonine	HRS	0.32 (0.05)	0.76 (0.12)	0.25 (0.26)	5.70 × 10^−23^	NA^b^
Cocaine	HRS	0.14 (0.06)	0.34 (0.09)	0.05 (0.04)	4.37 × 10^−13^	1.72 × 10^−8^
Ecgonine	HRS	0.04 (0.01)	0.12 (0.02)	0.03 (0.04)	4.79 × 10^−22^	NA^b^
Ecgonine methyl ester	HRS	0.10 (0.02)	0.25 (0.06)	0.06 (0.06)	7.62 × 10^−16^	2.27 × 10^−3^
Methamphetamine	HRS	0.38 (0.09)	1.79 (0.23)	1.34 (0.35)	4.23 × 10^−33^	1.45 × 10^−18^
THC-COOH	HRS	0.53 (0.14)	0.77 (0.09)	0.97 (0.33)	1.74 × 10^−9^	8.41 × 10^−8^
THC-OH	HRS	0.06 (0.11)	0.12 (0.16)	0.26 (0.13)	NA^b^	1.15 × 10^−7^

Abbreviations: ADI, Area Deprivation Index; DEET, N-diethyl-meta-toluamide; EDDP, 3-diphenylpyrrolidine; HRS, high risk substance; MDMA, 3,4-methylenedioxymethamphetamine; NA, not applicable; PPCP, pharmaceuticals and personal care products; RUCA, rural-urban commuting area; TCEP, tris (2-chloroethyl) phosphate; THC-COOH, 11-nor-9-carboxy-Δ-9-tetrahydrocannabinol; THC-OH, 11-hydroxy-Δ-9-tetrahydrocannabinol.

^a^ 2-sided *P* value.

^b^ NA because values are not statistically significant.

**Figure 4.**
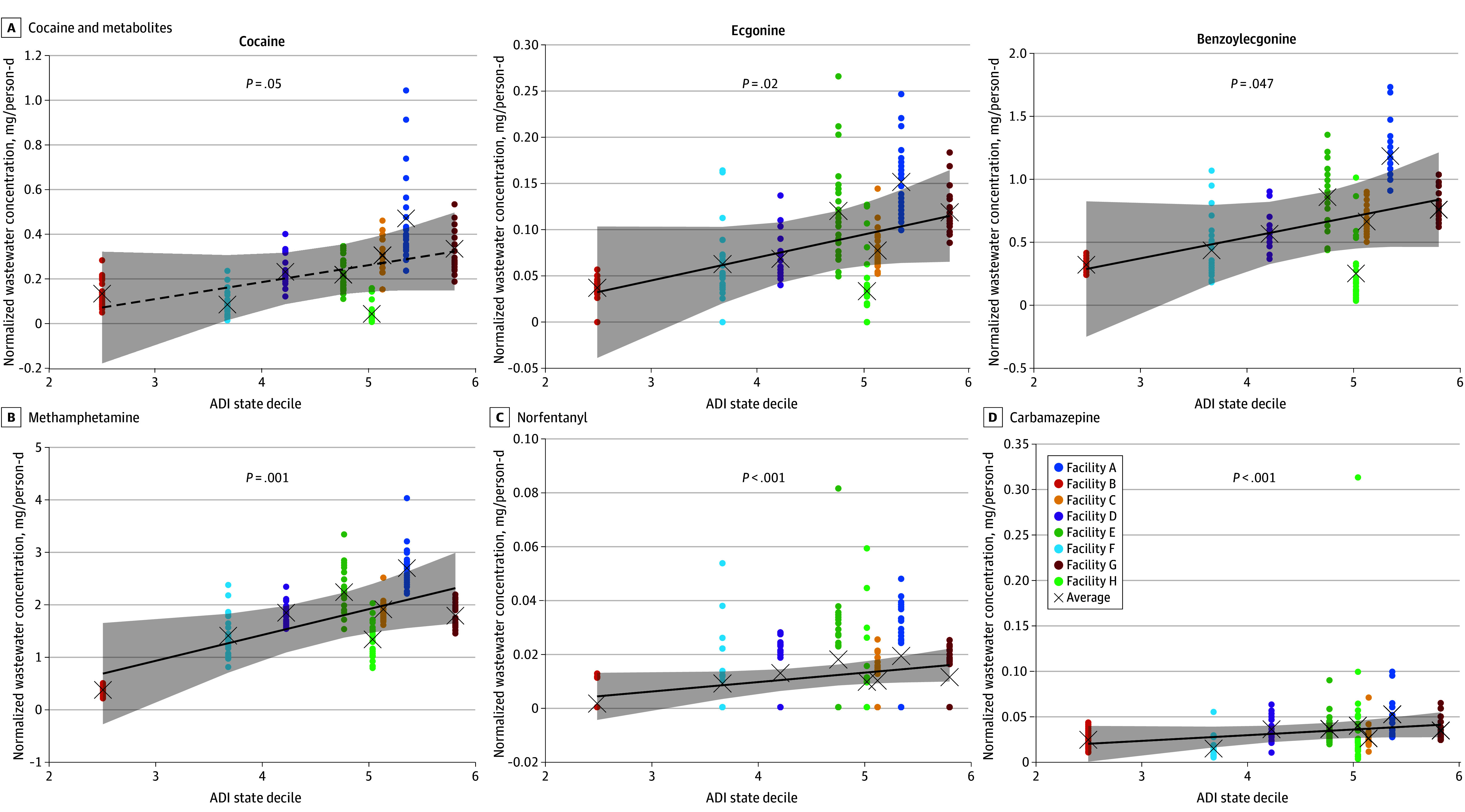
Association of Drug Use With Neighborhood Context Revealed by Area Deprivation Index (ADI) Increased drug use was associated with higher ADI (ie, more disadvantaged communities) for cocaine and its metabolites (A) and methamphetamine, norfentanyl, and carbamazepine (B). Circles represent normalized wastewater concentrations (mg/person-day) for individual samples collected from each sewershed, and crosses indicate the mean normalized wastewater concentration. Sewersheds were randomly anonymized as facilities A through H.

Among the 30 analytes that displayed a significant location or location × time effect in the LME model, (eFigures 2-4 in Supplement 1), the consumption and occurrence patterns of 6 analytes exhibited a significant positive correlation with the mean ADI of each facility (ie, more disadvantaged). This included cocaine (β = 0.075; SE = 0.038; *P* = .05) and its metabolites ecgonine (β = 0.025; SE = 0.011; *P* = .02) and benzoylecgonine (β = 0.167; SE = 0.083; *P* = .047) (Figure 4A), as well as methamphetamine (β = 0.493; SE = 0.149; *P* = .001); norfentanyl, a major metabolite of fentanyl (β = 0.004; SE=0.001; *P* = 1.64 × 10^−5^); and the anticonvulsant carbamazepine (β = 0.006; SE = 0.002; *P* = 3.59 × 10^−4^) (Figure 4B). These associations further underscore the relationship between drug use patterns in different facilities and socioeconomic factors.

## Discussion

Wastewater monitoring is an innovative tool for addressing the growing problem of drug abuse.^6,14,28^ In our longitudinal, cross-sectional study, we investigated the temporal and spatial patterns of 16 PPCPs and 18 HRSs across 8 sampling locations in southern Nevada from May 2022 to April 2023. Our analysis revealed significant temporal variations in drug consumption based on wastewater concentrations of target analytes, highlighting an overall increase in HRS use over time, alongside seasonally fluctuating PPCP utilization patterns. Moreover, by correlating wastewater drug consumption data with neighborhood contexts after anonymizing the facilities, we observed significantly greater HRS use in more disadvantaged areas, as determined by ADI. These findings underscore the potential for wastewater monitoring programs to not only serve as a reliable method for tracking drug consumption but also as a tool for identifying specific drug use patterns influenced by the sociodemographic and socioeconomic characteristics of communities.

The unsupervised clustering analysis of drugs in wastewater revealed large-magnitude correlations in estimated consumption patterns, specifically an increase in 4 cocaine-related analytes, between methadone and its derivative EDDP, and between methamphetamine and its partially excreted form, amphetamine (Figure 2A). These findings support the utility of wastewater data as a reliable source of information to determine drug exposure. Moreover, the clustering analysis of facilities highlighted geographically varied use patterns, aligning with geographic distances (eg, facility 4A and 4B) and sociodemographic similarities (eg, facility 1 and 3 serving large populations) across facilities, even without prior information on the neighborhoods they serve (Figure 1B). These findings imply that concentrations of drugs in wastewater collected from individual treatment facilities are influenced by distinct community characteristics, laying a foundation for further exploration of how community characteristics correlate with wastewater-derived drug use patterns.

Our LME analyses revealed a significant temporal increase in all 4 cocaine-related analytes over the 1-year duration of the study (Figure 2B), and a substantial increase at facility 1 over the last decade (eTable 6 in Supplement 1). These wastewater data align with the increase in cocaine-related emergency department visits and hospital admissions reported in the 2022 Nevada Epidemiologic Profile,^29^ underscoring the effectiveness and reliability of wastewater data in tracking drug consumption trends. Furthermore, consistent with previous studies associating cocaine use with income status in different geographic areas,^6,30,31^ our application of the state-ranked ADI to assess regional socioeconomic conditions (encompassing education, income, housing, and household characteristics) also supports these findings. Spatial analysis revealed that communities with lower socioeconomic standing tend to show higher wastewater-estimated cocaine use (Figure 4A), a pattern similarly observed in the increased use of another CNS stimulant, methamphetamine, in these more disadvantaged neighborhoods (Figure 4B).

Wastewater-estimated opioid use was highly correlated with the consumption of NSAIDs, marijuana, and CNS stimulants (Figure 2A), suggesting facility-level polydrug use often associated with chronic pain management. While opioid use increased significantly between 2010 and 2023 (eTable 6 in Supplement 1), only norfentanyl and hydrocodone showed temporal changes from 2022 to 2023 (Figure 2B), indicating relatively consistent use of most opioids over the 1-year duration of this study. This finding could also be attributed to the diversity of available drugs influencing the fluctuation in individual opioid consumption. The relatively stable pattern in marijuana use from May 2022 to April 2023, as revealed by the population-weighted average mass loadings of THC-COOH across all 8 treatment plants, further corroborated the relatively stable monthly cannabis sales data (based on cannabis tax revenue^32,33^) during the same period. A detailed analysis of norfentanyl trends, reported in Gerrity et al,^14^ showed a significant increase after October 2022, consistent with local clinical reports of fentanyl-related deaths in southern Nevada.^14^ Similar to cocaine, increased detection of norfentanyl was associated with neighborhoods facing socioeconomic disadvantages (Figure 4B), aligning with previous findings that higher prescription opioid rates correlate with social determinants of health, such as poverty, unemployment, lower education levels, and unstable housing, both in the US and internationally.^6,30,31^ Given these findings and the observed increase in HRSs in disadvantaged neighborhoods, combined with lifestyle challenges and limited health care resources in these areas,^34^ wastewater monitoring of HRSs could inform long-term public health planning in these communities.

Our LME analysis further revealed significantly different use patterns across facilities. Consistent with having the lowest ADI in this study (ie, least disadvantaged), facility B exhibited the lowest wastewater concentrations for nearly every HRS (Figure 4, Table, and eFigure 3 in Supplement 1), reinforcing the association of HRS use with neighborhood socioeconomic status. A closer look at facility H showed lower consumption of CNS stimulants and moderate use of opioids and marijuana (eFigure 3 in Supplement 1). Unique in its RUCA code of 2, these patterns in facility H suggest that drug use is influenced not only by socioeconomic factors but also by sociodemographic elements like urbanization.

Significant temporal changes were recorded in only 3 of the 16 PPCPs analyzed. In contrast with HRS use, most PPCP use, compared with data from 2010, remained stable, indicating generally consistent consumption over time (eTable 6 in Supplement 1). The use of DEET, an insect repellent, exhibited marked seasonal variation with a peak in summer months (Figure 2B), affirming the reliability of wastewater data for monitoring PPCP use. Additionally, the consumption of acetaminophen varied quarterly, with an increase during the holiday season, while antibiotics like trimethoprim showed significant bimonthly fluctuations (eFigure 1 in Supplement 1), suggesting regular and periodic use of these substances. Across different catchment areas, we observed significant variations in PPCP consumption and occurrence for all analytes except antibiotics (sulfamethoxazole and trimethoprim), TCEP (a flame retardant), and triclosan (an antimicrobial agent found in some soaps and lotions) (eTable 5 in Supplement 1). Interestingly, only the use of the anticonvulsant carbamazepine, like HRSs, showed a significant positive correlation with the ADI (Figure 4B), possibly due to its use in treating neuropathic pain.^6^ These patterns imply a relatively uniform use of PPCPs across socioeconomic strata, or alternatively, suggest that factors other than socioeconomic status, as indicated by ADI, may influence PPCP use in specific communities.

### Limitations

This study faces several limitations. First, the ADI offers refined resolution at 9-digit zip code levels, but the sewersheds served by our sampling locations span multiple 5-digit zip codes, leading to a generalized rather than precise socioeconomic status estimation for each facility. Although the RUCA codes are specific to 5-digit zip codes, uniform RUCA codes across all facilities simplified urbanization characterization. Notably, facilities 6 and 2, serving fewer zip code areas, provided a more reliable socioeconomic status context using ADI, reflected in the distinct consumption patterns of HRSs observed in these facilities. Second, our analysis assumes that drug concentrations in wastewater directly represent population consumption, but confounding factors such as method sensitivity limitations, in-sewer transformation, and alternative drug disposal methods may impact observed concentrations. Third, our LME model only captures linear associations of drug use with ADI scores or temporal changes, suggesting that more advanced multivariate and nonlinear methods could better assess complex associations. Finally, facility 1 serves the Las Vegas Strip, an area known for tourism attractions and hospitality. Due to the mixing of analytes from tourists and the local population, our current analysis of facility 1 is likely influenced by the confounding effects of mobile populations.

## Conclusions

To our knowledge, this is the first longitudinal, cross-sectional study to examine how spatiotemporal drug use behaviors, examined through community wastewater, can be integrated with ADI or RUCA scores in the US. The results of this study demonstrate how wastewater data can complement more traditional tools to provide an unbiased estimate of public health conditions within communities and the socioeconomic and demographic indicators that might influence public health and behaviors.

## Notes

Supplement 1. **eTable 1.** Characterization of Each Wastewater Treatment Plant (Average Daily Flow Rate in Million Gallons per Day [mgd]) and Corresponding Sewershed Population
eMethods.
**eTable 2.** Target Compound List With Associated Isotopes, Method Reporting Limits (MRLs), Multiple Reaction Monitoring (MRM) Transitions, and Electrospray Ionization (ESI) Mode **eTable 3.** Mass Spectrometer Source Parameters in Electrospray Ionization (ESI) Positive Mode **eTable 4.** High Risk Substance and Metabolite Compound List With Associated Isotopes, Method Reporting Limits (MRLs), and Multiple Reaction Monitoring (MRM) Transitions **eFigure 1.** Significant Temporal Trends Between May 2022 and April 2023 in the Linear Mixed Model for Six Analytes After False Discovery Rate (FDR) Correction **eTable 5.** F-Statistics and Significance Levels of Time, Facility (ie, Sewershed), and Time-Facility Interactions in the Linear Mixed Effects Model **eTable 6.** Direct Comparison of Flow- and Population-Normalized PPCP and HRS Concentrations (mg/d-Person) at Facility 1 During the Same Months in 2010 and 2023 **eFigure 2**. Usage Patterns of 12 PPCPs Exhibiting a Significant Location Effect in the Linear Mixed Effect Model **eFigure 3.** Usage Patterns of 16 HRS Analytes Exhibiting a Significant Location Effect in the Linear Mixed Effect Model **eFigure 4**. Temporal Usage Patterns Across Eight Sewersheds for Analytes Exhibiting a Significant Location x Time Effect in the Linear Mixed Effect Model
eReferences.

Supplement 2.
Data Sharing Statement
