# Supplementary material for: Drug Use Patterns in Wastewater and Socioeconomic and Demographic Indicators
Source: JAMA Netw Open. 2024 Sep 23;7(9):e2432682. doi: 10.1001/jamanetworkopen.2024.32682 (PMC11420698; doi:10.1001/jamanetworkopen.2024.32682)
Supplement: Supplement 1. — eTable 1. Characterization of Each Wastewater Treatment Plant (Average Daily Flow Rate in Million Gallons per Day [mgd]) and Corresponding Sewershed Population eMethods. eTable 2. Target Compound List With Associated Isotopes, Method Reporting Limits (MRLs), Multiple Reaction Monitoring (MRM) Transitions, and Electrospray Ionization (ESI) Mode eTable 3. Mass Spectrometer Source Parameters in Electrospray Ionization (ESI) Positive Mode eTable 4. High Risk Substance and Metabolite Compound List With Associated Isotopes, Method Reporting Limits (MRLs), and Multiple Reaction Monitoring (MRM) Transitions eFigure 1. Significant Temporal Trends Between May 2022 and April 2023 in the Linear Mixed Model for Six Analytes After False Discovery Rate (FDR) Correction eTable 5. F-Statistics and Significance Levels of Time, Facility (ie, Sewershed), and Time-Facility Interactions in the Linear Mixed Effects Model eTable 6. Direct Comparison of Flow- and Population-Normalized PPCP and HRS Concentrations (mg/d-Person) at Facility 1 During the Same Months in 2010 and 2023 eFigure 2. Usage Patterns of 12 PPCPs Exhibiting a Significant Location Effect in the Linear Mixed Effect Model eFigure 3. Usage Patterns of 16 HRS Analytes Exhibiting a Significant Location Effect in the Linear Mixed Effect Model eFigure 4. Temporal Usage Patterns Across Eight Sewersheds for Analytes Exhibiting a Significant Location x Time Effect in the Linear Mixed Effect Model eReferences. [file jamanetwopen-e2432682-s001.pdf]

Supplemental Online Content

Zhuang X, Moshi MA, Quinones O, et al. Drug use patterns in wastewater and socioeconomic and demographic indicators. *JAMA Netw. Open*. 2024;7(9):e2432682. doi:10.1001/jamanetworkopen.2024.32682

eTable 1. Characterization of Each Wastewater Treatment Plant (Average Daily Flow Rate in Million Gallons per Day [mgd]) and Corresponding Sewershed Population

eMethods.

eTable 2. Target Compound List With Associated Isotopes, Method Reporting Limits (MRLs), Multiple Reaction Monitoring (MRM) Transitions, and Electrospray Ionization (ESI) Mode

eTable 3. Mass Spectrometer Source Parameters in Electrospray Ionization (ESI) Positive Mode

eTable 4. High Risk Substance and Metabolite Compound List With Associated Isotopes, Method Reporting Limits (MRLs), and Multiple Reaction Monitoring (MRM) Transitions

eFigure 1. Significant Temporal Trends Between May 2022 and April 2023 in the Linear Mixed Model for Six Analytes After False Discovery Rate (FDR) Correction

eTable 5. F-Statistics and Significance Levels of Time, Facility (ie, Sewershed), and Time-Facility Interactions in the Linear Mixed Effects Model

© 2024 Zhuang X et al. *JAMA Network Open.*

eTable 6. Direct Comparison of Flow- and Population-Normalized PPCP and HRS Concentrations (mg/d-Person) at Facility 1 During the Same Months in 2010 and 2023

eFigure 2. Usage Patterns of 12 PPCPs Exhibiting a Significant Location Effect in the Linear Mixed Effect Model

eFigure 3. Usage Patterns of 16 HRS Analytes Exhibiting a Significant Location Effect in the Linear Mixed Effect Model

eFigure 4. Temporal Usage Patterns Across Eight Sewersheds for Analytes Exhibiting a Significant Location x Time Effect in the Linear Mixed Effect Model

eReferences

This supplemental material has been provided by the authors to give readers additional information about their work.

eTable 1. Characterization of Each Wastewater Treatment Plant (Average Daily Flow Rate in Million Gallons per Day [mgd]) and Corresponding Sewershed Population

	Average Daily Flow: million gallons per day	Sewer population: number of people
Facility 1	100	872,009
Facility 2	5	86,330
Facility 3	42	757,418
Facility 4	22	248,509
Facility 4A	16	133,977
Facility 4B	6	114,532
Facility 5	20	255,008
Facility 6	0.8	16,399

© 2024 Zhuang X et al. *JAMA Network Open.*

eMethods

Analytical Methods: A brief summary of methods used in previous publications (Gerrity et al., 2022[1] and 2024[2]) are described below:

1) Pharmaceuticals and Personal Care Products (PPCPs): Pharmaceuticals and personal care products (PPCPs) listed in **eTable 2** were analyzed using liquid chromatography tandem mass spectrometry (LC-MS/MS). Automated solid-phase extraction (ASPE) was employed for cleanup and concentration of target compounds. Samples were extracted using hydrophilic-lipophilic balance (HLB) cartridges, and the resulting extracts were concentrated and brought to a final volume using methanol. Chromatographic separation was achieved using a binary gradient of ammonium acetate in water and methanol, with electrospray ionization (ESI) in positive and negative modes. Method detection limits (MDLs) and method reporting limits (MRLs) were determined for each analyte.

2) High Risk Substances and Metabolites: High risk substances and metabolites listed in **eTable 3** were analyzed by direct injection LC-MS/MS after 1:10 dilution with reagent water. Isotopically labeled analogs were added to each sample for quantitation purposes. Separation was performed using a Raptor Biphenyl column with a gradient of formic acid in water and methanol. Mass spectrometry source parameters (**eTable 4)** were optimized for the target compounds. MDLs were determined using fortified reagent water, and MRLs were set at 3-5 times the MDL, adjusted for the dilution factor. Calibration curves were prepared for each analysis, ranging from 5-1,000 ng/L for most compounds and 10-2,000 ng/L for THC and its metabolites.

© 2024 Zhuang X et al. *JAMA Network Open.*

eTable 2. Target Compound List With Associated Isotopes, Method Reporting Limits (MRLs), Multiple Reaction Monitoring (MRM) Transitions, and Electrospray Ionization (ESI) Mode

Compound	Isotope	MRL (ng/L)	Q1 (m/z)	Q3 (m/z)	ESI Mode
Acetaminophen	d4-acetaminophen	100	150	107 (108)	Negative
Atenolol	d7-atenolol	20	267	145 (116)	Positive
Caffeine	d9-caffeine	100	195	110 (42)	Positive
Carbamazepine	d10-carbamazepine	5	237	165 (194)	Positive
DEET	d7-DEET	20	192	119 (91)	Positive
Fluoxetine	d5-fluoxetine	10	310	44 (148)	Positive
Gemfibrozil	d6-gemfibrozil	5	249	121 (127)	Negative
Ibuprofen	d3-ibuprofen	20	205	161 (159)	Negative
Meprobamate	d3-meprobamate	5	219	158 (97)	Positive
Naproxen	d3-naproxen	10	229	169 (185)	Negative
Primidone	d5-primidone	10	219	162 (91)	Positive
Sucralose	d8-sucralose	500	395 (397)	35	Negative
Sulfamethoxazole	d4-sulfamethoxazole	5	254	156 (92)	Positive
TCEP	d12-TCEP	200	285	99 (161)	Positive
Triclocarban	d8-triclocarban	40	313	160 (162)	Negative
Triclosan	d7-triclosan	20	287	35 (37)	Negative
Trimethoprim	d9-trimethoprim	5	291	261 (123)	Positive

( ) – confirmation product ions; Abbreviations: DEET = N,N-diethyl-meta-toluamide; TCEP = Tris(2-chloroethyl) phosphate.

© 2024 Zhuang X et al. *JAMA Network Open.*

eTable 3. High Risk Substance and Metabolite Compound List With Associated Isotopes, Method Reporting Limits (MRLs), and Multiple Reaction Monitoring (MRM) Transitions

Compound	Isotope	MRL (ng/L)	Q1 (m/z)	Q3 (m/z)
6-Acetylmorphine	d6-6-acetylmorphine	50	328	165 (211)
Amphetamine	d8-amphetamine	100	136	91 (119)
Benzoylecgonine	d8-benzoylecgonine	50	290	168 (105, 82.3)
Cocaine	d3-cocaine	50	304	182 (82, 105)
Codeine	d6-codeine	50	300	152 (115)
EDDP	d3-EDDP	50	278	234 (186, 219)
Ecgonine	d3-EME[a]	100	186	100.3 (168)
EME	d3-EME	50	200	82 (182)
Heroin	d9-heroin	100	370	165 (268)
Hydrocodone	d6-hydrocodone	50	300	199 (128)
MDA	d5-MDA	100	180	105 (133, 77)
MDMA	d5-MDMA	100	194	163 (135, 77)
Methadone	d9-methadone	50	310	105 (265)
Methamphetamine	d8-methamphetamine	100	150	91 (119)
Morphine	d6-morphine	50	286	152 (165)
Norcocaine	d3-norcocaine	50	290	168 (136)
Norfentanyl	d5-norfentanyl	50	233	84 (150)
Oxycodone	d6-oxycodone	50	316	241 (256)
THC	d3-THC	1,000	315	193 (123)
THC-COOH	d9-THC-COOH	1,000	345	193 (299)
THC-OH	d3-THC-OH	1,000	331	193 (201, 313)
Tramadol	d3-tramadol	50	264	58 (42.2)

( ) – confirmation product ions; Abbreviations: EDDP = 2-ethylidene-1,5-dimethyl-3,3-diphenylpyrrolidine; EME = ecgonine methyl ester; MDA = 3,4-methylenedioxyamphetamine; MDMA = 3,4-methylenedioxymethamphetamine; THC = delta-9-tetrahydrocannabinol; THC-OH = 11-hydroxy-delta-9-tetrahydrocannabinol; THC-COOH = 11-nor-9-carboxy-delta-9-tetrahydrocannabinol; [a]d3-ecgonine is not available so ecgonine quantitation is based on the next closest isotope with respect to structure and retention time, which is d3-EME in this method.

© 2024 Zhuang X et al. *JAMA Network Open.*

eTable 4. Mass Spectrometer Source Parameters in Electrospray Ionization (ESI) Positive Mode

**Parameter**	**Value**
Curtain gas (CUR)	20
Collision gas (CAD)	12
Ion spray voltage (IS)	5500
Ion source gas 1 (GS1)	60
Ion source gas 2 (GS2)	50
Temperature (TEM)	550
Entrance potential (EP)	10

© 2024 Zhuang X et al. *JAMA Network Open.*

eFigure 1. Significant Temporal Trends Between May 2022 and April 2023 in the Linear Mixed Model for Six Analytes After False Discovery Rate (FDR) Correction

(A) increase in the cocaine metabolites ecgonine, benzoylecgonine, and ecgonine methyl ester; **(B)** increase in hydrocodone; and **(C)** seasonality in acetaminophen and trimethoprim.

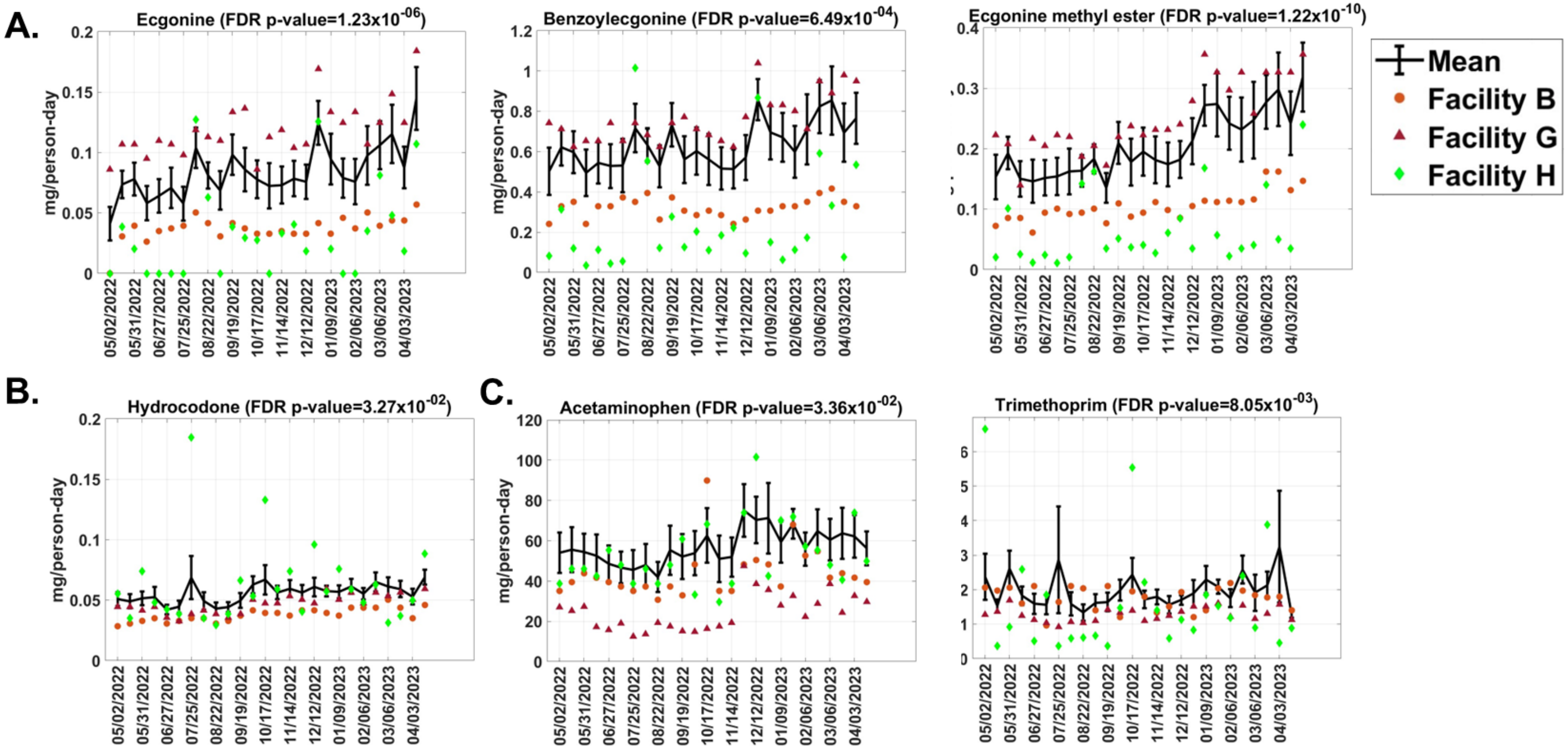

© 2024 Zhuang X et al. *JAMA Network Open.*

eTable 5. F-Statistics and Significance Levels Of Time, Facility (i.e., Sewershed), and Time-Facility Interactions in the Linear Mixed Effects Model

Significance levels (p-values) were corrected for multiple comparisons using the false discovery rate (FDR) method, and only FDR-adjusted p-values <0.05 are listed. Degrees of freedom (dF) are listed below each F-statistic.

Name	Category	Detailed Category	Fvalue: Facility dF(7,72)	FDR-p: Facility	Fvalue: Time dF(1,72)	FDR-p: Time	Fvalue: Interaction dF(7,72)	FDR-p: Interaction
Acetaminophen	PPCP	NSAID	21.63	3.70E-20	6.13	3.36E-02		
Ibuprofen	PPCP	NSAID	20.84	1.44E-19				
Naproxen	PPCP	NSAID	24.63	1.76E-22				
Atenolol	PPCP	Licit Drug	14.34	5.10E-14				
Carbamazepine	PPCP	Licit Drug	5.91	1.31E-05			3.22	8.79E-03
Fluoxetine	PPCP	Licit Drug	10.04	6.36E-10				
Gemfibrozil	PPCP	Licit Drug	2.72	2.64E-02				
Meprobamate	PPCP	Licit Drug	3.04	1.25E-02				
Primidone	PPCP	Licit Drug	2.63	3.18E-02				
Sulfamethoxazole	PPCP	Licit Drug						
Trimethoprim	PPCP	Licit Drug			9.31	8.05E-03		
Caffeine	PPCP	Daily	3.63	3.57E-03				
DEET	PPCP	Daily	6.62	2.28E-06	5.37	4.87E-02		
Sucralose	PPCP	Daily	3.19	8.89E-03				
TCEP	PPCP	Daily						
Triclosan	PPCP	Daily						
Acetylmorphine	HRS	Opioid	3.24	8.52E-03				
Codeine	HRS	Opioid	2.87	1.89E-02				
EDDP	HRS	Opioid	14.22	6.21E-14				
Hydrocodone	HRS	Opioid	8.78	1.14E-08	6.22	3.27E-02		
MDMA	HRS	Opioid	5.83	1.55E-05				
Methadone	HRS	Opioid	26.03	1.86E-23				
Morphine	HRS	Opioid	3.77	2.61E-03			3.19	8.89E-03
Norfentanyl	HRS	Opioid			62.20	1.66E-12	4.52	4.16E-04
Oxycodone	HRS	Opioid	15.53	4.72E-15				
Tramadol	HRS	Opioid	17.22	1.68E-16				
Amphetamine	HRS	Stimulant	48.84	2.21E-37			2.51	4.03E-02
Benzoylecgonine	HRS	Stimulant	16.85	3.22E-16	14.62	6.49E-04		
Cocaine	HRS	Stimulant	14.84	1.88E-14	50.59	1.40E-10	3.29	7.99E-03
Ecgonine	HRS	Stimulant	10.00	6.66E-10	28.48	1.23E-06	3.57	4.06E-03
Ecgonine methyl ester	HRS	Stimulant	10.60	1.77E-10	51.09	1.22E-10	4.78	2.23E-04
Methamphetamine	HRS	Stimulant	45.26	1.05E-35				
THC-COOH	HRS	Marijuana	28.34	4.72E-25				
THC-OH	HRS	Marijuana	9.06	6.12E-09				

© 2024 Zhuang X et al. *JAMA Network Open.*

Supplementary Table 6. Direct Comparison Of Flow- And Population-Normalized PPCP and HRS Concentrations (mg/day-Person) at Facility 1 During the Same Months in 2010 and 2023.

Green, increase in consumption between 2010 and 2023 for 14 HRS; yellow, the level of PPCP usage in 2023 paralleled those in 2010; red, declined usages in 2023 as compared to 2010.

Changes in usage were computed by: $\frac{\text{(Average of Columns 6 and 7)} - \text{(Average of Columns 5 and 6)}}{\text{(Average of Columns 5 and 6)}} \times 100.$

Drug	PPCP/HRS	Drug category	2/7/2010	3/7/2010	2/6/2023	3/6/2023	Change in usage
Acetaminophen	PPCP	OTC Pain Reliever			86.82	91.16	
Ibuprofen	PPCP	NSAID			11.72	13.46	
Naproxen	PPCP	NSAID			7.81	8.68	
Atenolol	PPCP	Licit Drug	0.79	0.78	0.61	0.78	-11.87%
Carbamazepine	PPCP	Licit Drug	0.04	0.04	0.07	0.06	48.25%
Fluoxetine	PPCP	Licit Drug			0.03	0.03	
Gemfibrozil	PPCP	Licit Drug			0.61	0.61	
Meprobamate	PPCP	Licit Drug	0.35	0.34	0.06	0.08	-80.51%
Primidone	PPCP	Licit Drug	0.08	0.05	0.10	0.10	59.58%
Sulfamethoxazole	PPCP	Licit Drug	0.40	0.46	0.41	0.48	3.19%
Trimethoprim	PPCP	Licit Drug	0.28	0.29	0.23	0.23	-21.25%
Caffeine	PPCP	Daily			47.75	56.43	
DEET	PPCP	Daily	0.07	0.08	0.08	0.20	78.17%
Sucralose	PPCP	Daily			43.41	56.43	
TCEP	PPCP	Daily	0.13	0.17			
Triclosan	PPCP	Daily					
Acetylmorphine	HRS	Opioid			0.02	0.02	
Codeine	HRS	Opioid			0.07	0.07	
EDDP	HRS	Opioid			0.07	0.07	
Hydrocodone	HRS	Opioid			0.06	0.08	
MDA	HRS	Opioid	0.02	0.02			
MDMA	HRS	Opioid	0.12	0.11	0.05	0.07	-46.60%
Methadone	HRS	Opioid				0.02	
Morphine	HRS	Opioid	0.26	0.30	0.32	0.25	2.08%
Norcocaine	HRS	Opioid	0.01	0.01			
Norfentanyl	HRS	Opioid			0.04	0.04	
Oxycodone	HRS	Opioid			0.06	0.06	
Tramadol	HRS	Opioid			0.16	0.17	
Amphetamine	HRS	Stimulant	0.13	0.14	0.30	0.31	128.17%
Benzoylecgonine	HRS	Stimulant	0.81	0.55	1.13	1.48	90.78%
Cocaine	HRS	Stimulant	0.34	0.33	0.48	0.65	69.11%
Ecgonine	HRS	Stimulant	0.31	0.30	0.13	0.17	-48.89%
Ecgonine methyl ester	HRS	Stimulant	0.18	0.15	0.40	0.52	175.47%
Methamphetamine	HRS	Stimulant	0.92	1.04	2.69	2.78	180.21%
THC-COOH	HRS	Marijuana			1.95	2.04	
THC-OH	HRS	Marijuana			0.65	0.78	

© 2024 Zhuang X et al. *JAMA Network Open.*

eFigure 2. Usage Patterns of 12 PPCPs Exhibiting a Significant Location Effect in the Linear Mixed Effect Model

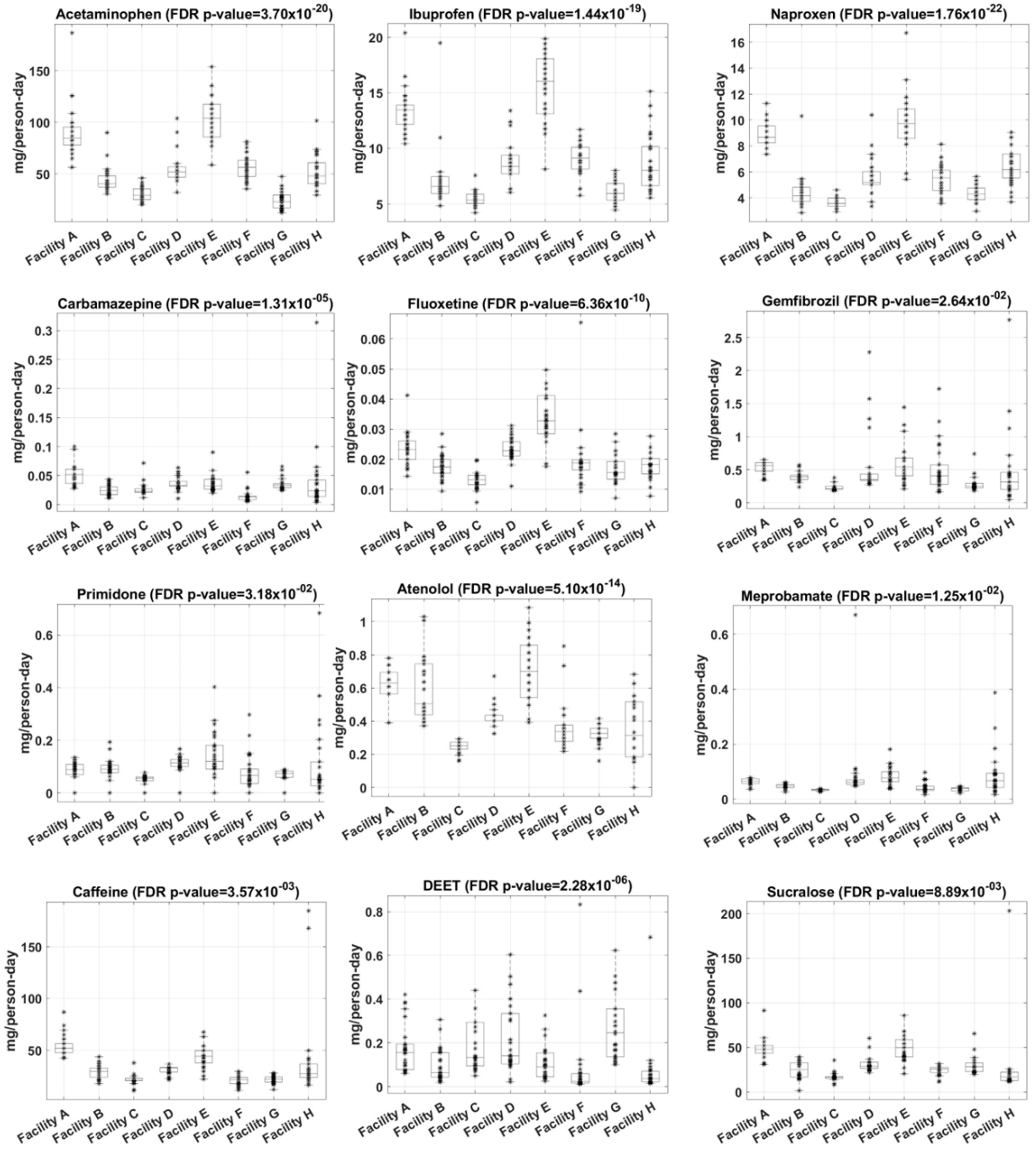

© 2024 Zhuang X et al. *JAMA Network Open.*

eFigure 3. Usage Patterns of 16 HRS Analytes Exhibiting a Significant Location Effect in the Linear Mixed Effect Model

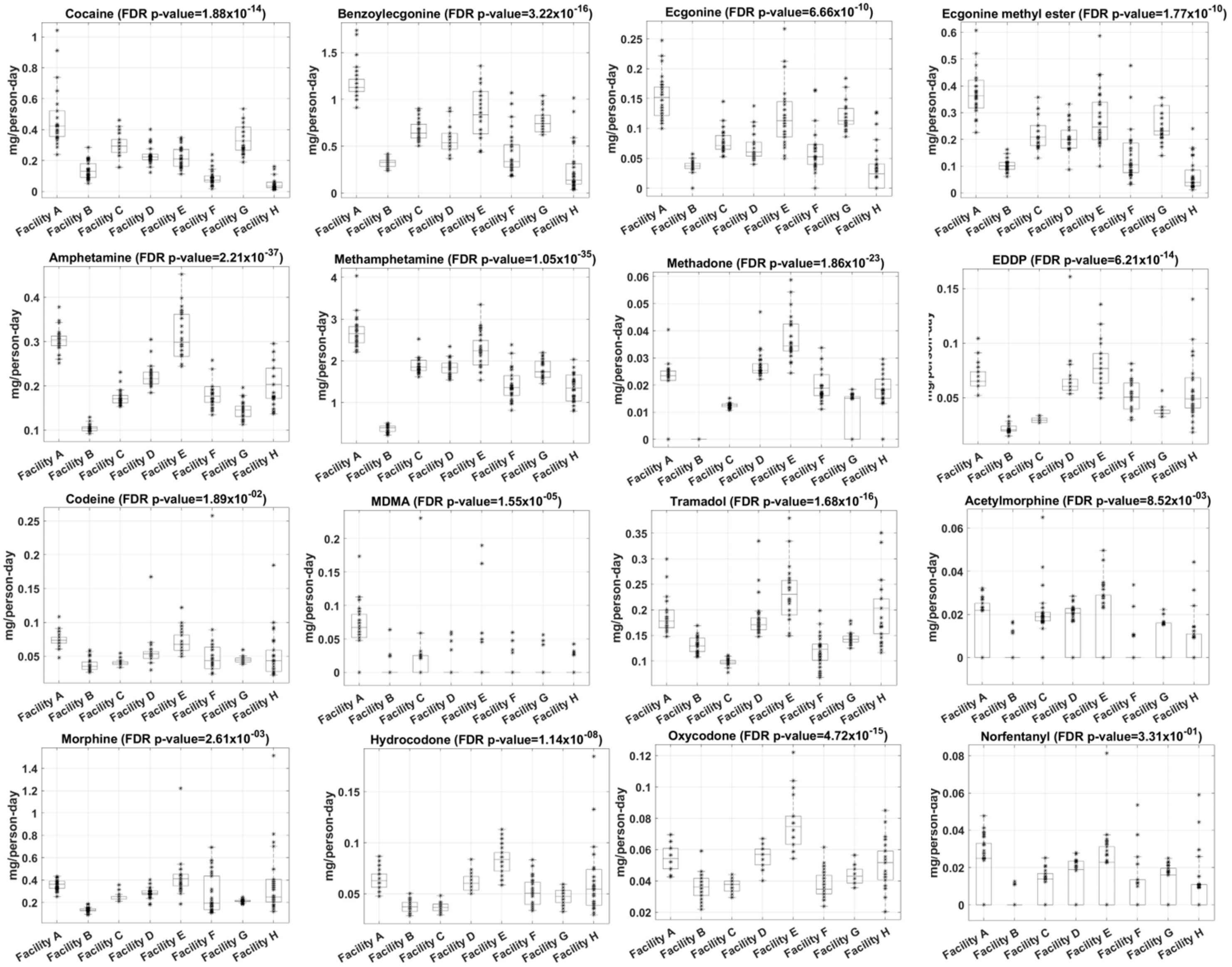

© 2024 Zhuang X et al. *JAMA Network Open.*

eFigure 4. Temporal Usage Patterns Across Eight Sewersheds for Analytes Exhibiting a Significant Location x Time Effect in the Linear Mixed Effect Model

(A) four stimulants/metabolites, **(B)** two opioids/metabolites, and **(C)** one PPCP

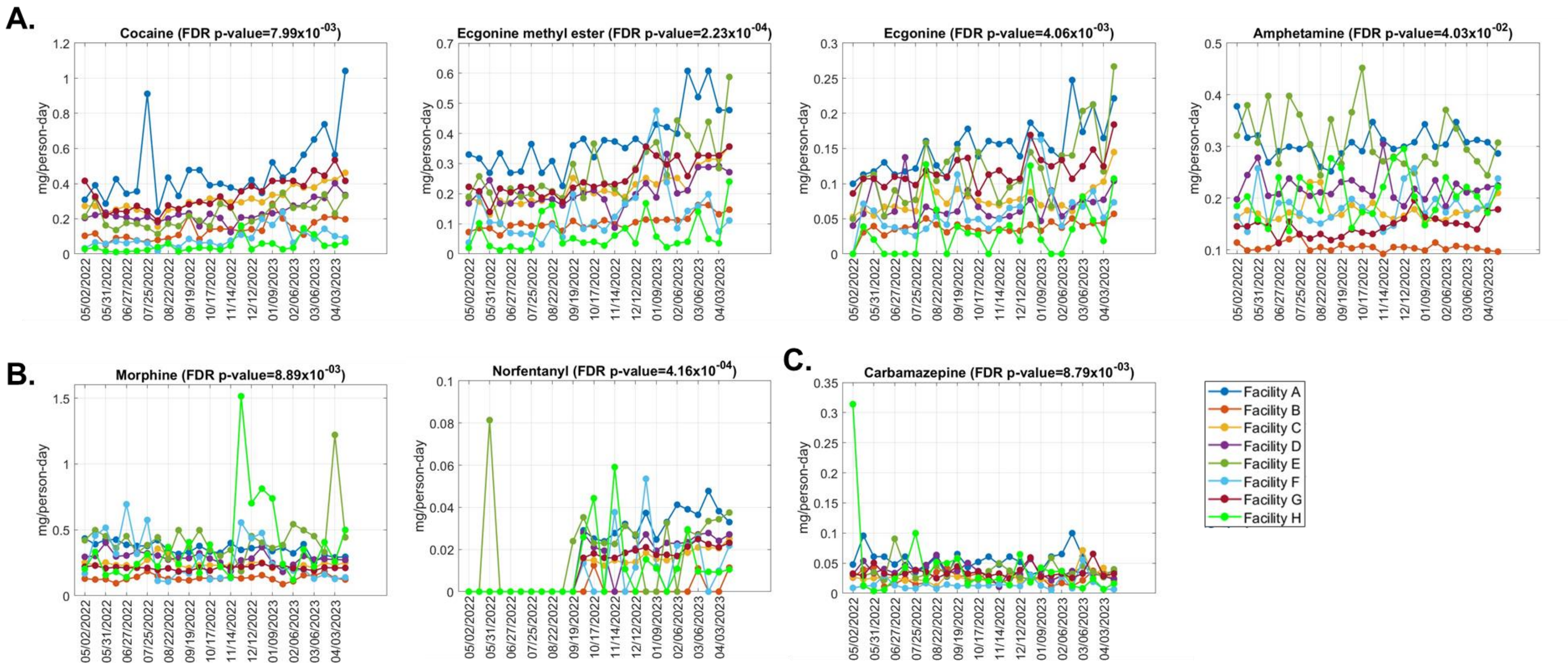

© 2024 Zhuang X et al. *JAMA Network Open.*

eReferences

1. Gerrity D, Papp K, Dickenson E, et al. Characterizing the chemical and microbial fingerprint of unsheltered homelessness in an urban watershed. *Science of The Total Environment*. 2022;840:156714. doi:10.1016/J.SCITOTENV.2022.156714

2. Gerrity D, Crank K, Oh EC, Quinones O, Trenholm RA, Vanderford BJ. Wastewater surveillance of high risk substances in Southern Nevada: Sucralose normalization to translate data for potential public health action. *Science of The Total Environment*. 2024;908:168369. doi:10.1016/j.scitotenv.2023.168369

© 2024 Zhuang X et al. *JAMA Network Open.*
