# Supplementary material for: Drug Use Patterns in Wastewater and Socioeconomic and Demographic Indicators
Source: JAMA Netw Open. 2024 Sep 23;7(9):e2432682. doi: 10.1001/jamanetworkopen.2024.32682 (PMC11420698; doi:10.1001/jamanetworkopen.2024.32682)
Supplement: Supplement 2. — Data Sharing Statement [file jamanetwopen-e2432682-s002.pdf]

Data Sharing Statement

Zhuang. Drug Usage Patterns Correlate With Wastewater and Socioeconomic and Demographic Indicators. *JAMA Netw Open*. Published September 23, 2024. doi:10.1001/jamanetworkopen.2024.32682

Data
Data available: Yes
Data types: Data (not involving human participants)
How to access data: edwin.oh@unlv.edu
When available: With publication

Supporting Documents
Document types: None

Additional Information
Who can access the data: anyone requesting the data
Types of analyses: for any purpose
Mechanisms of data availability: with or without investigator support
Any additional restrictions: None
